# Evaluation of Optical Detection Platforms for Multiplexed Detection of Proteins and the Need for Point-of-Care Biosensors for Clinical Use

**DOI:** 10.3390/s141222313

**Published:** 2014-11-25

**Authors:** Samantha Spindel, Kim E. Sapsford

**Affiliations:** Division of Biology, Chemistry, and Materials Science; Office of Science and Engineering Laboratories; U.S. Food and Drug Administration, 10903 New Hampshire Avenue, Silver Spring, MD 20993, USA; E-Mail: kim.sapsford@fda.hhs.gov

**Keywords:** immunoassay, fluorescent, ELISA, multiplex, protein, biomarker, point-of-care, *in vitro* diagnostics, quantum dot

## Abstract

This review investigates optical sensor platforms for protein multiplexing, the ability to analyze multiple analytes simultaneously. Multiplexing is becoming increasingly important for clinical needs because disease and therapeutic response often involve the interplay between a variety of complex biological networks encompassing multiple, rather than single, proteins. Multiplexing is generally achieved through one of two routes, either through spatial separation on a surface (different wells or spots) or with the use of unique identifiers/labels (such as spectral separation—different colored dyes, or unique beads—size or color). The strengths and weaknesses of conventional platforms such as immunoassays and new platforms involving protein arrays and lab-on-a-chip technology, including commercially-available devices, are discussed. Three major public health concerns are identified whereby detecting medically-relevant markers using Point-of-Care (POC) multiplex assays could potentially allow for a more efficient diagnosis and treatment of diseases.

## 1. Introduction: Multiplexed Biomarker Detection for Clinical Needs

Technological advances in fields such as genomics, proteomics and metabolomics have advanced our understanding of the underlying mechanisms of disease initiation, disease progression, and therapeutic response, and helped identify biomarkers useful in personalized medicine [1–3]. These biomarkers, such as proteins, can serve as diagnostic, prognostic, or therapeutic indicators and typically represent a surrogate endpoint used in addition to, or instead of, a clinical endpoint. Development of novel devices for biomarker measurement is important to the field of personalized medicine, a term which is defined in numerous ways. For the purpose of this review, personalized medicine is defined as providing the best treatment specific to a patient's individual genomic, or proteomic profile to guide safer and more effective treatment [4–6]. Information about a patient's make-up on a cellular level can provide clues regarding the appropriate medication, pertinent drug dosage, disease state, or method for disease prevention [4]. The ultimate goal of personalized medicine is to achieve the “5 Rs”: (1) the right patient; (2) right diagnosis; (3) right treatment; (4) right drug/target; and (5) right dose/time. Such a goal can only be realized through the combination of a clinical approach to medicine, completion of a comprehensive medical history, and utilization of data from appropriate testing such as *in vitro* diagnostic devices. While the 5Rs represent ideals of healthcare, there are many issues with arriving at the right diagnosis in practice as these elements rely upon the accuracy of tests and prevalence of disease present. In fact, positive and negative predictive values are more important for clinicians than the sensitivity and specificity of the test [7].

*In Vitro* Diagnostics (IVDs) are assays that probe samples taken from a patient (urine, blood, nasal swabs, *etc.*) for molecular, genomic, epigenomic, or proteomic species to aid in the clinical diagnostics, prognostics or in treatment selection [8]. Specific IVDs that are developed in parallel with a therapeutic agent, and are used in conjunction with one another as specified on the labeling of the drug and device, are called companion diagnostics [6]. Specific analytes that IVDs probe are considered “biomarkers” if they can be objectively measured and evaluated and indicate normal or pathogenic biologic processes or pharmacologic responses to a particular therapeutic intervention [6,9]. There are three major categories of biomarkers—(1) biomarkers of exposure (e.g., diagnosis/identification of disease or to predict response to therapy); (2) susceptibility (e.g., to distinguish patients with indolent or aggressive disease); and (3) toxicity (e.g., to identify patients likely to develop adverse side effects) [10]. Ideally, IVDs should be high-throughput, rapid, and capable of real-time detection of multiple biomarkers. IVDs for personalized medicine should also be paired with a specific drug or drug combination that would be able to treat the patient safely and effectively with minimal adverse effects [11].

### 1.1. Significance of Multiplexed Protein Detection

The scientific community has a growing interest in multiplexing (simultaneous detection of multiple analytes), which has developed within the last decade [11]. Multiplexing is important because disease and therapeutic response often involve the inter-play between many biological processes, and hence proteins rather than a single entity [11]. DNA and proteomic microarrays have been crucial in identifying new biomarkers and will continue to play a significant role in their routine detection [4,5,12–16]. Many efforts have been made to understand the biological basis of disease by studying gene expression, but the relationship between expression of a gene and the onset of disease remains unclear. The relationship between protein profiles and disease onset, however, is becoming better understood [17]. As there are only around 25,000 genes in the human genome and genes code for multiple variants of proteins, researchers often use proteomics, rather than genes, to provide insight into diseases [18] and new analytical tools can assist in this process [5,6]. Multiplexing is important for immunoassays, for example, a biochemical technique that uses antibodies for measuring the amount of a specific macromolecule present in a sample. Since antibodies are created due to the body's immune response against viruses, bacteria, and other hazards, using these biological molecules for detection purposes achieves superb specificity and sensitivity [18]. Concentrations as low as 10^−21^ moles/L have been detected using immunoassays [18]. Multiplexing can increase throughput and increase data generation while simplifying formats and decreasing the time and cost required to operate tests [19].

### 1.2. Importance of Multiplexing for Clinical Use

Many scientific fields can benefit from the use of multiplexed sensors, but the focus of this review is to interrogate the development of multiplexed biosensors for clinical use or personalized medicine. The need for the development of a multiplexed biosensor for use in clinical practice is paramount for therapeutic and diagnostic purposes where it is important to both identify and quantify biomarkers (analytes that indicate normal or pathogenic biologic processes) [20]. Measuring many different proteins at the same time is useful because one biomarker may be indicative of more than one disease, related diseases can manifest with similar physical symptoms, and monitoring diseases requires detection of subtle differences over time [21].

Multiplexed sensors have the potential to revolutionize patient treatment by reducing exposure to potentially ineffective toxic drug treatments based on the assessment of many biomarkers [22]. Furthermore, multiplexed sensors can help prevent disease and prolong life if: a diagnosis is made early, patients are screened multiple times over a specific period, and only the best therapies are prescribed for patients.

The standard techniques for protein multiplexing are not currently suited for a Point-of-Care (POC) environment. Detection of analytes in low volumes is particularly relevant for tissue biopsies for which volumes may be limited. In clinical laboratories, 0.5 mL of serum is required for antibody studies, and 0.15 mL is needed per assay [23]. For some critically ill, neonatal, or pediatric patients, the amount of serum needed may be prohibitive for analysis. However, sample size is less of an issue for multiplex assays if they can be miniaturized and used at a POC.

### 1.3. Benefits of POC Biosensors in the Healthcare Field

A POC device is useful in the healthcare field because it can be used to confirm a patient's diagnosis (for example, confirm a diagnosis based on a clinical exam) while the patient is still present in the doctor's office. Rapid confirmation of a clinical finding by implementing a quantitative test is particularly important if the patient is contagious or requires immediate treatment. For example, standard laboratory tests for detecting sexually transmitted infections typically take 2–14 days until results are obtained, whereas a 20-min POC test is expected and a 5-min turnaround time for a POC test is ideal [24]. A model POC device would require a single test sample, a single set of personnel to process and analyze the sample and a single point in time when the patient's blood or urine would need to be sampled. These benefits can help increase the likelihood of a patient to follow through for repeat care or to find out the outcome of a test, thereby improving the level of care provided.

In clinical practice, determining the presence of biomarkers typically requires a variety of laboratory tests and equipment that utilize several different types of technology. For a single diagnosis, many laboratories and personnel can be involved in handling each test. The more laboratories and personnel needed to analyze samples for a single diagnosis, the greater the uncertainty associated with the results. Typical assays such as an Enzyme-Linked Immunosorbent Assay (ELISA) allow single analyte detection, but multiplexed POC devices can be used to probe for many biomarkers simultaneously using one uniform testing method, thereby allowing for greater consistency in the data obtained for each analyte. Furthermore, determining relationships between biomarkers is easier and more reliable when using the same testing method and sample as in a multiplexed POC device. Multiplexing increases the speed of detection and quantification is simpler because signals from samples can be directly compared to background noise and controls located on the same device. Multiplex POC assays require less time, cost, and labor, and smaller sample size; the sample size is often irrespective of the number of analytes tested and only dependent on the detection technique used [25].

## 2. Sensor Requirements for POC Use

A biosensor is defined by the International Union of Pure and Applied Chemists (IUPAC) as a self-contained device that provides quantitative analytical information using a biological recognition element such as an antibody or nucleic acid, which is in direct contact with a transduction element [11]. In other words, biosensors use a biological system to differentiate substances of interest from other components in a sample by employing a biological receptor to detect the analyte, a transducer to convert the recognition event to a signal, and a detection system that includes analysis and processing [18]. The ideal POC device is small, portable, cost-effective, highly accurate, low maintenance, easy to use, robust, and stable under different environmental conditions [23]. The device should also require minimal user interface, have a rapid turnaround time, and high-throughput capability. The specifications of POC devices are key for the integration of all aspects of an assay (sampling, testing, and detection) onto one hand-held platform to create a true biosensor [26]. Device attributes such as Limit of Detection (LOD), specificity, sensitivity, reproducibility, reliability, robustness, cost, speed, and multiplexing capability are crucial in the design of a biosensor that can be used in a POC environment.

Appropriately-designed IVDs can be particularly useful as POC diagnostics, which may be used in a doctor's office or at home and can reduce the time, sample volume, and reagent volumes required as well as the overall cost of the test. Since many POC IVDs measure one analyte, are homogeneous and simple to use (e.g., glucose test strips or home pregnancy tests), they often require only one mixing/sample addition step and one detection step [8,27,28]. Therefore, there is potential for these types of assays to become miniaturized and incorporated into high-throughput, multiplex devices POC devices. As currently designed, standard techniques involving microtiter plates are not suited for a POC environment because they are complicated, requiring multiple steps and trained personnel to operate.

### 2.1. Small Sample Volume

The use of such small volumes (hundreds of μL) is important for medical practice because large volumes (a few mLs) of sample may not be obtainable from certain patients, such as infants or the critically ill [20,21,25] or from all sample types, such as tissue biopsy or cerebrospinal fluid [29–34]. Intake of small sample volumes (100 μL) could enable the use of capillary blood taken from the finger of a patient rather than venous blood, requiring a phlebotomist's assistance. Furthermore, when a patient's kidneys are damaged, the production of urine may be inhibited, so performing a test on a small volume of fluid can be useful [35].

A standard 96-well microtiter plate immunoassay such as an ELISA requires 50–100 μL of sample or reagent per well [36]. However, miniaturized immunoassays have been conducted using microscope glass slides in many formats, including sandwich and reverse phase formats, and these platforms typically require a sample volume in the high micro-to-milliliter range for operation [37–40].

For home care or other “point of use” applications, the device must be relatively small in size (not much larger than a cellular phone); thus the sample volume required to detect analytes should be ≤50 μL. However, sample size is less of an issue for multiplex assays, which can be miniaturized. Efforts to miniaturize assays have involved microfluidics, lab-on-a-chip technologies, and improved excitation and detection methods [8,27,28,41].

Small sample size confers an important advantage in that there may be less influence from matrix effects on the assay when experiments are conducted in biological fluids such as plasma and urine [18]. Different sample sizes, each containing the analyte concentration, may provide different results. However, in small samples, there is a lower proportion of the sample in the reaction, so the influence of matrix effects may be diminished and the accuracy improved. Therefore, POC assays using small sample sizes may be more sensitive compared to conventional methods. However, small sample size may also pose a risk as it is also possible that analytes may be undetected, depending upon the concentration.

### 2.2. Detection System

Two commonly-used fluorescent assays capable of multiplexed protein detection are standard microtiter plate fluorescent assays and the Luminex 100/200. These standard optical methods of multiplexed protein detection often require large instrumentation, highly-trained personnel, and must be performed in a typical laboratory setting [42,43]. In contrast, POC devices have many advantageous attributes such as their low maintenance and portability. Although miniature ELISAs and chip-based flow cytometry have been demonstrated as a proof-of-concept, no such devices have been commercialized, so the need to develop a POC biosensor is still paramount [44–46].

In order to create a smaller detection system, the method for analyzing the signal of biomarkers for immunoassay platforms could be coupled to a charge-coupled device (CCD) or complementary metal-oxide-semiconductor (CMOS) camera, especially for a fluorescent detection system [47]. In this setup, either a light-emitting diode (LED) or a small laser diode could be used as the excitation source, but the LED may be more advantageous rather than a laser, which would have to move over a large distance in order to illuminate the entire surface, unless the laser causes total internal reflection. If the device is portable, disposable, cost-effective, requires minimal user interaction, and meets a clinical need to obtain relevant information about analytes in small volumes [23], physicians could make diagnostic, prognostic, or therapeutic decisions more quickly than with existing technology [21]. The detection system size can range from the size of a cell phone (˜14 cm × 7 cm × 1 cm) to the size of a pen (˜15 cm × 1 cm × 1 cm), depending upon the technology used. Some POC devices require connection to a computer screen, making them larger.

Microfluidics and lab-on-a-chip technology can be applied to PoC applications to enable miniaturization, automation, and integration of assay components (e.g., detection) to create handheld devices [8,26]. Microfluidics have been demonstrated for use in immunoassays, for example, with antibody-immobilized beads for detection of analytes [48]. Automation, whether by use of microfluidic technology, or other technology increases the performance, robustness, and reliability of assays [49]. The ideal POC device would combine the experimental procedures and the detection systems into one integrated, fully-fledged, self-contained device that requires minimal user interaction and that features a digital readout to assist with interpretation of results. Lateral flow tests such as the pregnancy test, which is considered by many patients and clinicians to be the best POC prototype, represents an easy-to-use, easy-to-read POC device that combines sampling and detection [7,24].

## 3. Protein Detection

Currently, there are many different methods for detecting proteins in a multiplexed fashion such as through electrochemical, photometric, mechanical [50], or piezoelectric [51] means. Some biosensors are composed of nanomaterials [52], while others use microcantilevers [53], yet all methods of detection involve either label-based or label-free techniques [54]. Optical sensors [55], for example surface plasmon resonance [56] or mass spectroscopy [57,58], are advantageous over many other detection strategies because they are immune to interferences from electrochemical and electromagnetic sources, are capable of real-time detection [59], and are amenable to lab-on-a-chip formats. Current issues with these biosensing strategies include less than optimal limits of detection, low sensitivity, and low specificity relative to the demands of certain applications. In addition, many biosensors are unable to detect different analytes simultaneously in an automated fashion [51]. The gold standard for protein detection is ELISA, which can measure multiple targets in different wells (*i.e.*, spatial separation) using colorimetric, Raman, fluorescent, or luminescent techniques [11]. There are two main types of multiplexing strategies—Spatial separation on a surface (*i.e.*, different wells or spots) and the use of unique identifiers/labels (*i.e.*, spectral separation due to different colored dyes or bead sizes/colors).

A number of the current multiplex methods for protein detection rely on immunoassays. An immunoassay is a biochemical technique that uses antibodies for measuring the amount of a specific macromolecule present in a sample. Some of the advantages of immunoassays are that they can be used to detect antigens indicative of disease with great specificity and sensitivity [18]. However, immunoassays are prone to cross-reactivity among capture antibodies, particularly when target antigens are present at different concentration ranges [11]. Immunoassays may also have decreased sensitivity due to matrix effects. Biotin, an essential component of vitamin B, and avidin, a protein commonly found in albumin [60], are routinely used as part of the assay in a number of immunoassay and other techniques, typically during the detection step(s) or in competitive assay formats [61–63]. The avidin-biotin complex is one of the strongest known non-covalent interactions (Ka ˜1015 M-1) and is often used for sensing applications [64–67]

**Figure 1.**
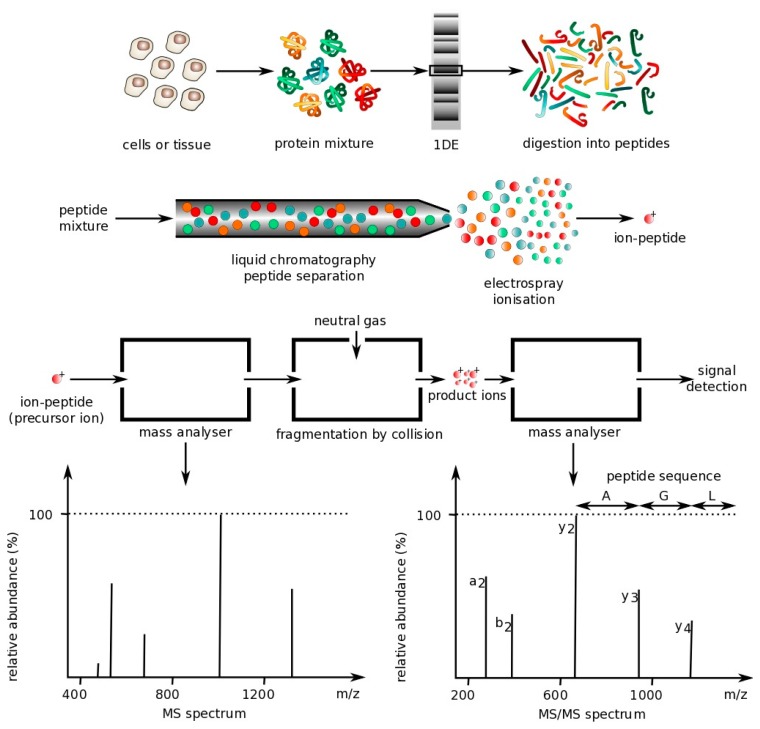
Mass spectrometry protocol [74] under license Philippe Hupé/CC-BY-SA-3.0.

The following sections describe the main optical techniques used for protein multiplexing. However, one technique commonly used is mass spectrometry, which is not an optical technique. Mass spectrometry is a technique that can be used to analyze protein mixtures and quantify thousands of proteins [68]. Measurements are conducted in the gas phase of ionized analytes. Mass spectrometers are composed of three components: An ion source to ionize analytes, a mass analyzer for measuring ionized analytes' mass-to-charge ratio (m/z), and a detector that counts the number of each m/z ion. The two most common techniques for volatizing and ionizing proteins are (1) electrospray ionization (ESI), which ionizes analytes from a liquid-based sample and (2) matrix-assisted laser ionization (MALDI), which ionizes analytes from a dry matrix, with the former used more commonly for complex samples. This direct technique can achieve high specificity, but tends to have a lower sensitivity, requires large laboratory equipment and is expensive to operate, making it not ideal for a POC environment [18]. However, some recently-described hand-held mass spectrometry units have been reported that operate autonomously with the use of a wireless remote control or demonstrate similar analytical performance to large-scale mass spectrometers, but do not require any experimental setup [69,70]. These field-deployable mass spectrometers have application in detection of explosives and uranium (e.g., following a nuclear accident) as well as analysis of compounds in complex biological samples [69,71,72], but have not yet been applied to detection of large peptides and proteins [73]. Below is a figure that demonstrates how mass spectrometry works (Figure 1).

### 3.1. Conventional Optical Detection Methods for Multiplexed Protein Detection

#### 3.1.1. Surface Plasmon Resonance (SPR)

**Figure 2.**
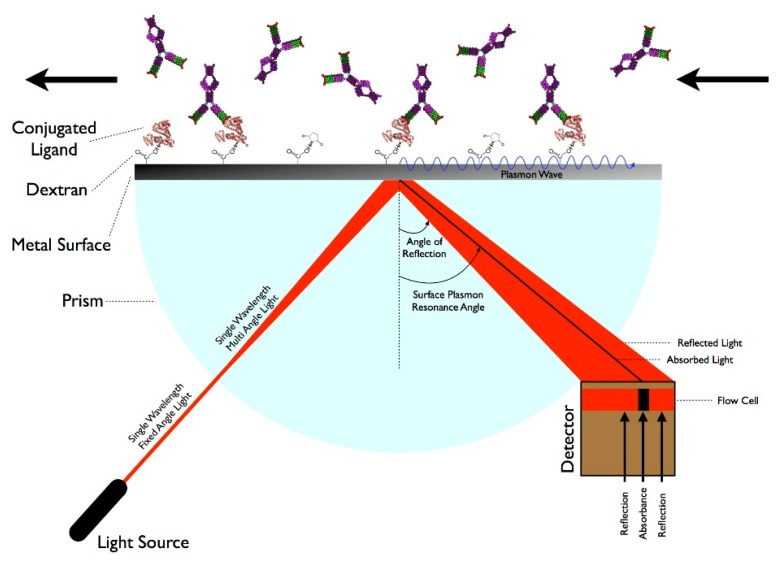
Surface Plasmon Resonance (SPR) [81] under license Sara Sabban/CC-BY-SA-3.0.

There are many techniques that involve measuring molecular interactions using a label-free format including Quartz Crystal Microbalance, BioLayer Interferometry, and Resonant Waveguide Grating, but SPR is the leading technology [18]. SPR is a technique that measures specific molecular interactions—such as the binding of proteins to antibodies—where the analyte in solution interacts with the molecules bound (e.g., via carboxymethylated dextran polymers) to the sensor surface, usually composed of a thin-gold film on a glass surface [75]. The gold side of the sensor surface is in contact with a flow channel while the glass side of the sensor surface is positioned on a prism. Under total internal reflection, light transforms photons into surface plasmons contained in the gold layer. Light must hit the surface at a specific angle of incidence, which depends upon the refractive index in the proximity to the gold surface, in order to be reflected and reduced, generating a characteristic surface plasmon resonance (SP) band. When analytes bind to the surface, the change in mass concentration causes a shift in the refractive index, which in turn shifts the SP band and the amount of light absorbed by the detector. The angle of incidence is monitored so that detection of binding events occurs in real-time. While this technique has advantages of being label-free and relatively sensitive, this technology typically requires laboratory-based equipment due to the size of the optical components and highly-trained personnel, so it is not suitable for a POC environment [76]. However, some recent advances in label-free biosensing and the use of fibers or waveguides indicate that in-the-field SPR sensing may be on the horizon. Some examples include development of a compact SPR sensor capable of analyzing chemical contaminants in water and creation of a combined SPR imaging sensor and protein array for detection of proteins in plasma [77,78]. Other SPR devices have been developed to rapidly detect DNA and proteins in microliter volumes, suggesting that miniaturized SPR devices could be developed in the future [79,80]. Figure 2 demonstrates how SPR works.

#### 3.1.2. Flow Cytometry

Flow cytometry is a technique that measures biological-labeled beads as they pass through a detector in a fluid stream, one at a time [82]. These beads are used for multiplexing by either changing the internal label used (different dyes) or the size of the beads. Flow cytometry allows fluorescent molecules associated with particles to be distinguished from free fluorescent molecules. This technique is employed in a variety of clinical applications ranging from measuring cellular DNA content to identifying disease-specific cell types for diagnostic and prognostic purposes [42,83]. Flow cytometry is also used to perform immunoassays, with wide potential in detection of various biomarkers. There are many commercialized products capable of performing bead-based flow cytometry in a multiplexed fashion (e.g., Luminex products) by performing a sequential analysis on particles. Beads have been demonstrated as a means for sorting cells, proteins, or other particles of varying sizes [42]. Standard beads with specific fluorescence intensity can be used for establishing quality control—Data from samples taken over time and among different experiments can be normalized. Although flow cytometry can be used for diagnostic purposes, it is desirable to combine this technique with DNA analysis or other procedures to improve the value of flow cytometry [83]. Nevertheless, some advantages of this technique include its speed, accuracy, low background signal, reproducibility, cost-effectiveness, and sensitivity, although the size of typical commercial instruments may limit the application of flow cytometry to a POC environment [54]. However, microflow cytometers that incorporate small optical components and employ microfluidic technology have been developed and are portable [84]. Microflow cytometers have been applied to many fields such as characterization of marine algae and detection of bacteria and toxins in clinical samples [85,86]. Also in development are lab-on-a-chip flow cytometers, but none are currently available for commercial purchase [45,46].

#### 3.1.3. Enzyme-Linked Immunosorbent Assay (ELISA)

**Figure 3.**
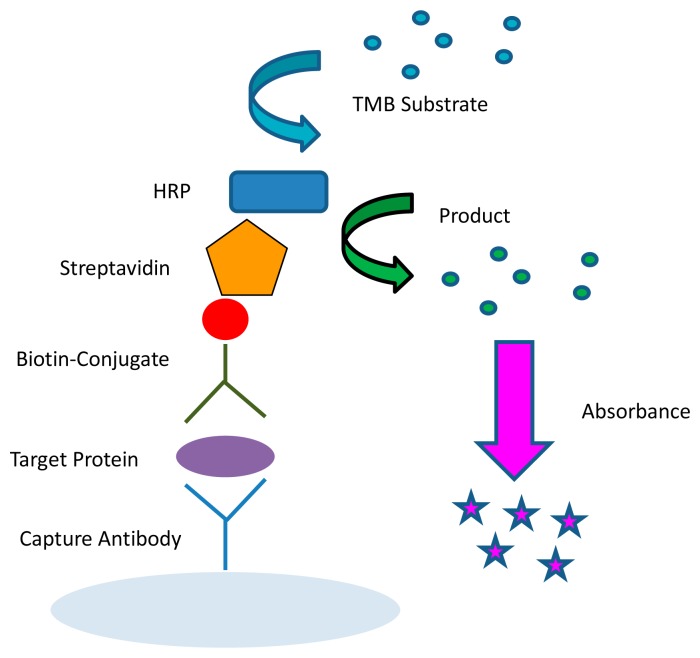
ELISA detection scheme using the enzyme horseradish peroxidase (HRP).

The most commonly used and best validated method to quantify biological molecules is ELISA (Figure 3) [11]. This method is capable of multiplexing by using spatially distinct wells that can probe for different proteins. The ELISA typically uses either a colorimetric or luminescent method for detection and causes a product of the colored reaction to absorb or produce light in the detectable range [87]. In colorimetric ELISAs, the optical density of the product is proportional to the amount of analyte measured. These assays are typically conducted using a 96-well microtiter plate. A similar method to the ELISA is the Meso Scale^®^™ system, which uses an electrochemiluminescent method for multiplexing. The Meso Scale can probe for different analytes within one single well in addition to among several different wells on a single plate.

ELISAs have some drawbacks with regard to their performance. When Coenen *et al.*, evaluated six different ELISAs for detection of the same target antibodies, the performance cutoff values, reproducibility, sensitivity, specificity, and accuracy for the detection of antibodies varied significantly, demonstrating the need for standardization among manufacturers' diagnostic tests [88]. Specifically, reagents, assays, data storage, and normalization techniques need to be standardized [25] due to the variability among similar assays.

ELISAs also have limited applicability when it comes to POC requirements. Obtaining data from ELISAs usually necessitates a dedicated laboratory and an expensive and bulky plate reader. However, Sapsford *et al.*, demonstrated the miniaturization of colorimetric SEB ELISAs, reducing sample volume to <5 μL/well [44]. There are also some ways to decrease user interaction with experiments such as using a sequential injection analysis technique, which can automate the washing and addition of reagent solutions with the use of a syringe pump and switching valve [89]. Recently, a new technique called digital ELISA has been introduced where sub-femtomolar concentrations of proteins can be detected [18]. In this single-molecule immunoassay, a solution of enzymes are trapped in 50-femtoliter wells containing fluorogenic substrates, along with the sample, capture beads, and detection antibody, and sealed. Digital ELISA avoids reliance on diffusion, as in standard 96-well experiments. While this technology is still in its infancy, digital ELISA is very promising for automated, high-throughput applications requiring single-molecule sensitivity. While ELISA would also be prone to matrix effects, the enzyme amplification is generally more sensitive than a fluorescent sandwich immunoassay, which lacks the amplification step [90].

#### 3.1.4. Fluorescence Immunoassays

ELISAs are similar to fluorescent immunoassays in that they are normally conducted in a sandwich format (two antibodies specifically bind to different epitopes on a common target) [91]. However, ELISAs use an enzyme, such as horseradish peroxidase (HRP), coupled with a colorimetric or chemiluminescent substrate for signal generation, instead of a fluorescent label [92,93]. In fluorescent immunoassays, the relative fluorescence units (number of photons emitted) are proportional to the amount of analyte present [87]. Fluorescent immunoassays may not require use of the biotin-streptavidin interaction, but instead expose a dye-labeled antibody to the target protein for detection, decreasing the amount of time it takes to perform an assay.

##### Fluorescent Detection Labels

A variety of fluorescent labels are available for use with fluorescent immunoassays. Organic fluorophores (fluorescent compounds that may re-emit light following excitation) such as dimeric cyanine dyes, Cy3 and Cy5 are commonly used in the literature for quantifying proteins [94,95]. Both dyes are often used because of their brightness, low non-specific dye interactions, and because they are commercially available with a wide range of reactive chemistries that facilitate labeling, such as the ability to label protein lysine residues [54]. Although fluorescent dyes are frequently used, they have limitations such as low photostability and brightness, and intrinsic background fluorescence [96].

**Figure 4.**
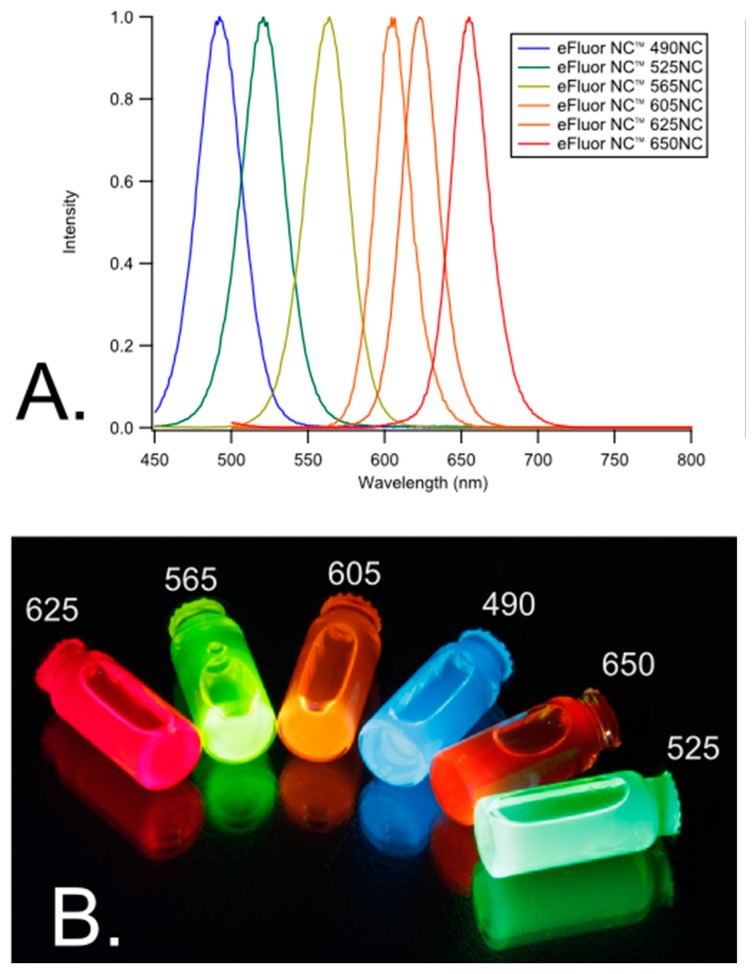
Properties of CdSe/ZnS quantum dots. (**A**) photoluminescence spectra; (**B**) quantum dots after UV excitation Adapted, with permission from [19]. Copyright (2011) American Chemical Society.

One multiplexing approach that combines both spatial and spectral multiplexing can be achieved using quantum dots (QD)s (Figure 4). This technique can increase the multiplexing capabilities for immunoassays because multiple QDs have broad absorption bandwidths and can be excited at one particular wavelength, yet they each have a narrow spectral bandwidth within the visible range. Therefore, the signal from each QD can be distinguished from one another.

Experiments using 96-well microtiter plates succeeded in deconvoluting signals from three different QDs corresponding to three target analytes [19]. These accomplishments indicate that, for example, a 96-well plate can yield more than 96 data points because more than one target analyte can be detected per well. There is promise to extend these studies to distinguish between even more analytes by employing more than three QDs so long as their emission wavelengths are spectrally diverse. QDs can be applied to both planar and suspension biochips, can be used to detect single molecules, and can be employed in fluorescence resonance energy transfer (FRET) for added specificity and POC use [96].

Some drawbacks of QDs are their elevated cost in comparison to other fluorescent labels, their toxicity, and their size [97]. A way to avoid the expense associated with purchasing QDs is to synthesize them in-house [98]. Since QDs are typically composed of heavy metals such as cadmium or lead, which are highly toxic materials, they may pose concern when applied for *in vivo* applications [97,99]. Lastly, QDs are typically larger in size than organic fluorophores, and this may disrupt the natural binding kinetics of proteins that are involved in the biosensing platform, so assays involving QDs must be optimized carefully.

### 3.2. Miniaturized Assays

Besides the clinical relevance for using small sample volumes (discussed in Section 2.1), there are other advantages of using low volumes such as simplification of the platform format, an increase in sensitivity, and an increase in throughput and subsequent volume of data [19]. For example, some microfluidic heterogeneous immunoassays have been shown to detect bacterial toxins with a LOD in the femtomolar range [100]. The LOD of one miniaturized immunoassay to detect autoantibodies implicated in autoimmune diseases reportedly attained 1/50th of the LODs of a classical ELISA and require 100-fold less volume [101]. Other studies involving autoantibody arrays have shown four to eight times higher sensitivity than ELISAs and were linear over a 1000-fold range [25]. These experiments spotted antigens onto the surface using a robotic arrayer and were probed with monoclonal antibodies or serum samples.

The use of protein array technologies for the analysis of diseases, while not a new concept, is emerging as a powerful technique for profiling protein levels and hence identifying biomarkers indicative of disease [5,35,36,102]. Although many modified ELISAs exist as commercial antibody assays, the kits often require many washing steps, produce a lot of waste, are expensive, and involve lengthy processes [103]. Despite these challenges, there are also issues related to the use of microarrays such as technical problems related to printing and detection, normalization of data, lack of reference samples between experiments and laboratories, as well as the ability to measure biomarkers that exist in samples at such varied concentrations [104].

Microscope glass slide arrays for multiplex detection of proteins have been developed previously, but the volumes needed to perform assays are generally around 50 μL [76]. There are multiplexed platforms for protein detection that have been developed by research groups and there are some commercially-available biosensors employing planar waveguide technology, but there is still potential for the reduction of volumes required to conduct these assays [105–112].

#### 3.2.1. Protein Microarrays

Protein microarrays have many benefits over traditional ELISA and fluorescence immunoassays (performed in 96-well plates) because they are helpful for determining antibody reactivity to a large number of targets using a relatively small amount of sample and in some cases have been reported to achieve better sensitivity [113,114]. They have been used for determining vaccination response, screening for disease-related biomarkers, and evaluating specificity of antibodies. However, compared to DNA arrays, protein arrays are not as precise or reproducible [17]. Many research groups have used antibody microarrays for biomarker multiplexing and compared their results to standard ELISAs [97]. While many journal articles address the potential of their platforms to be developed into POC systems, rarely are the reported systems actual stand-alone biosensors.

**Figure 5.**
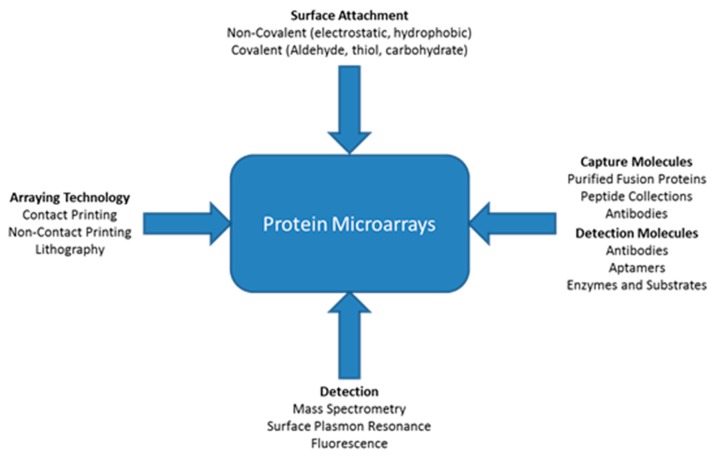
Different technologies for protein microarrays.

A variety of different types of technologies can be applied for protein microarrays. Figure 1 shows that there are multiple ways proteins can attach to a surface such as non-covalent or covalent attachment [115]. Different types of molecules can be immobilized onto the surface including antibodies, peptides, or purified proteins. There are three main types of immunoassay strategies for detection of proteins: Sandwich, antigen capture, and direct. There are many technologies that can be used in conjunction with appropriate buffers to keep the immobilized proteins in their most active state. The figure also shows various methods for detection, with some better than others for detection of low concentrations of proteins within small sample volumes. The detection method is important because there are no protein amplification procedures [115]. ELISAs can amplify the detection signal by employing an enzyme, but this does not amplify the protein itself. Based on the particular application and type of analysis desired, certain combinations of arraying technology, immobilization technique, capture molecules, and detection techniques (Figure 5) may be more suitable than others. With the right blend of microarray technologies, platforms can be designed to perform better than ELISA.

Protein microarrays are divided into two main groups: Planar surface assays or bead/suspension assays [49]. The former can be conducted on glass, silicon, or nitrocellulose, and uses solid-phase kinetics. The latter uses micron-sized beads that may be distinguished by color code, shape, or size by using an instrument such as a Luminex. Bead assays use fluid-phase kinetics, which may allow for faster detection than using planar surface assays.

Within planar surface arrays, there are two main types of quantitative protein microarrays: forward and reverse arrays [17]. Forward protein arrays involve immobilizing antibodies onto a surface and assessing the protein levels in samples that bind to the immobilized antibodies. On the other hand, reverse phase protein arrays bypass the need for a capture antibody because extracts from clinical samples are printed onto the surface which is then probed with proteins or antibodies [116]. These types of assays are often used to assess phosphorylation status [17]. Reverse phase assays have relatively low sensitivity compared to forward phase assays because when a protein is present at a low concentration, less proteins of interest bind to the surface compared to others, so primary antibodies have fewer binding sites available. However, reverse phase assays are only limited by the availability of high-affinity antibodies [116]. Since reverse phase formats rely on a single antibody, extensive validation is necessary for this type of assay. Reverse phase assays are common for autoantibody profiling [21].

Some multiplex protein microarrays require quantification of the signal while for other applications, a qualitative response is sufficient. For example, Rowe-Taitt *et al.* demonstrated the use of an assay to detect six different biohazardous agents using a format that requires fluorescent detection. Upon imaging, results can be interpreted by eye to determine whether or not an analyte is present (*i.e.*, if the spot on the assay probing for a specific analyte is bright or dull) [117]. In this case, simply knowing whether an agent is present is sufficient and the concentration of the agent is immaterial.

Some commercial protein microarrays have been developed, such as the AtheNA Multi-Lyte test system and the BioPlex 2200 ANA screen, which use Luminex's xMAP technology [49]. These immunoassays can screen for multiple autoantibodies that are involved in rheumatic diseases. The CombiChip Autoimmune is another commercially-available product that can be used to help identify autoimmune diseases; this product uses nitrocellulose-coated slides and requires manual imaging and analysis. Meso Scale Diagnostics employs an electrochemiluminescent method and can be used to quantify cytokines, chemokines, phosphoproteins, and toxicologic biomarkers, among many others.

##### Biological Molecule Immobilization

As mentioned, there are a variety of methods for immobilizing biological molecules onto different surfaces. Molecules may become immobilized in two main ways: by physical adsorption or through covalent bonds [118,119]. One common method for immobilization is through nonspecific physical absorption; this technique, however, is not as effective and reproducible as employing covalent binding [17]. Covalent binding is advantageous because only certain reactive groups are involved in forming bonds. Primary amines on the amino acids lysine and arginine are frequently used as the reactive group for binding due to their common presence on essentially all proteins. The most commonly-used amine-reactive chemistry is N-hydroxysuccinimide (NHS). 1-Ethyl-3-(3-dimethyl-aminopropyl)carbodiimide (EDC) is also frequently used because it is reactive with the carboxyl group present on the surface. However, this method is prone to issues such as hydrolysis of EDC in aqueous environments, susceptibility to cross-linking, and the requirement for more than 1000-fold excess of reagent, which can cause mixed avidity and heterogeneous architecture [19]. Materials such as plastics or silicon, as well as slides that are coated with nitrocellulose polymers, e.g., FAST slides [20,21,101,104] may be used in place of the chemistries previously described. Silicon surfaces are also promising [120,121] and can be modified physicochemically to bind proteins with an affinity comparable to FAST slides [122]. In addition, there are a number of different commercial slide surfaces, designed specifically for use with robotic microarrayers [20]. Antibodies may be patterned onto the surface using PDMS flow cells, stencils or by printing with robotic machines [123].

##### Drawbacks of Microarrays

There are a number of areas of concern when developing multiplex platforms for biomarker screening, with reproducibility (inter- and intra-slide variation) and sample normalization being the major concerns. In order to address some of the issues associated with protein arrays [20,21,40], a variety of controls can be implemented to allow broad market application of microarrays such as replicate spots, negative control spots, marker spots for orientation, and spots to test cross-reactivity of capture and detection antibodies [49].

Other factors that can affect the utility of microarrays include the use of polyclonal or monoclonal antibodies. Choosing the most appropriate antibody for experiments is important as the affinity and specificity can be affected. In addition, the conditions under which patterning of antibodies takes place such as the temperature and humidity must be tightly controlled. Lastly, blocking the surface with a blocking buffer/agent, such as PBS + 1% Bovine Serum Albumin, following patterning is critical to prevent non-specific adsorption of proteins.

In addition to assay variability, some microarrays exhibit less-than-optimal linear dynamic range [113]. Current methods for analyzing data typically rely upon the direct comparison of signal intensities, which limits quantification between antibodies and the fluorescent signal. Microarrays do not have a common standard for detecting antibodies that bind to different targets, as opposed to ELISA, which can utilize an independent standard curve. Since the affinity of antibodies for their targets can vary in biological matrices, methods are needed to quantify independent antibody concentrations in microarrays. A nonlinear calibration was developed to quantify the amount of antibody binding to the surface. This method adds a series of known amounts of antibodies (from the same species) onto an array. IgG is used to create a nonlinear standard curve, which is used to interpolate the amount of antibody that specifically binds to the epitope of each protein.

In addition to the drawbacks inherently associated with conducting protein microarrays, there are drawbacks with commonly used microarray scanners that are used to quantify results. For example, using the GenePix 4000B Microarray Scanner for analysis is problematic because the lasers used for excitation are confined to either the green or red range and the blue range is preferred for analyzing microarrays that use QDs. The laser power settings are limited to only 100%, 33%, or 10%. Another drawback is that the filter emission cannot be configured with each type of label used because the microarray scanner is optimized for only Cy3 and Cy5. Microarray scanners, like plate readers, are not optimized for POC use. The microarray scanner mentioned has dimensions of 34.29 cm × 20.32 cm × 44.45 cm and a weight of 25 lbs. and costs > $ 50,000. As a result, development of data detection platforms that are designed specifically for use with a certain fluorescent label are becoming more popular. Researchers can devise platforms that are configured to employ the proper lasers and filters to administer the most appropriate excitation wavelength and to capture the best range of emission wavelengths. However, in doing so, this may limit the application to a particular set of fluorophores.

#### 3.2.2. Lab-on-a-Chip Devices

Lab-on-a-Chip devices integrate processing steps such as sampling, sample pre-treatment, separation, detection, and data analysis into one small machine useful for POC sensing. Ideally, a Lab-on-a-Chip device should meet several specifications. First, the optic, electronic and fluidic components of the device must be incorporated in separate compartments so that the function of the optics and electronics is not impaired by exposure to fluid [124]. Second, the optics components should be incorporated into the device in a manner that makes replacing parts easy. Third, the reservoirs for fluid should be amenable for injection molding, as required for mass production. Fourth, the reservoirs for the sample and tracer molecules must be compartmentalized so that they do not mix. Microfluidic systems are capillary networks (typically 10–50 μm deep and 10–400 μm wide) fabricated on materials such as silicon, glass, or polymeric substrates [48]. Flow of fluid is usually controlled by electroosmotic effects such as application of an electric field or vacuum. Microfluidics can allow for parallelization and integration of sample processing steps onto one small device [125]. Other benefits include miniaturization, automation, and disposable units for single-use devices.

Microfluidic systems have been used in protein separation, kinase reactions, and immunoassays [48]. Sandwich assay formats, which require addition of the analyte, followed by a labeled antibody, have been demonstrated in microfluidic systems using glass or polystyrene beads that are immobilized and entrapped with samples containing the analyte of interest. In one example, when a syringe pump was used to control the flow, the reaction time was 30 min for four reaction chambers contained on a 50 mm × 70 mm space. In another example, a “SlipChip” was developed for conducting immunoassays using magnetic beads [126]. This approach involved two microfabricated glass slides with various inlets, outlets, and wells where the sample was exposed to the reagents required.

**Figure 6.**
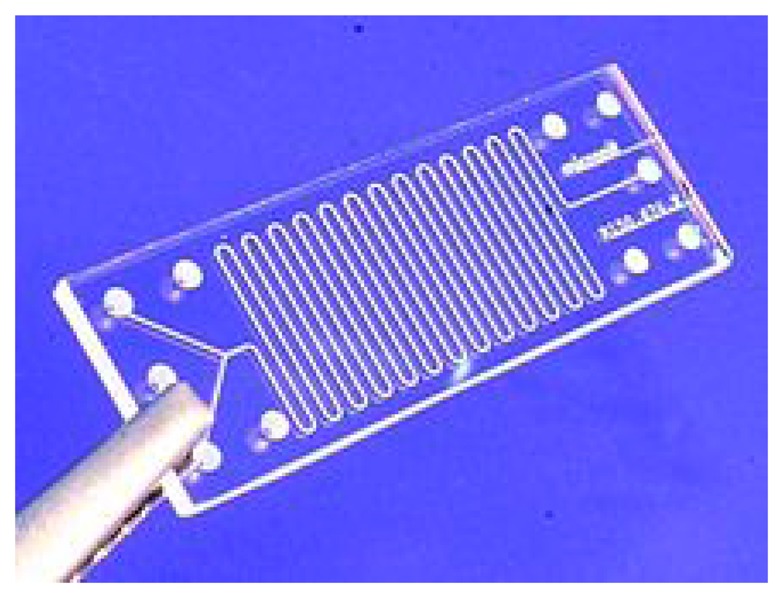
Lab-on-a-chip made of glass [127] under license © Micronit/Wikimedia Commons/CC-BY-SA-3.0.

Unfortunately, microfluidic systems have rarely matured from the proofs-of-concept in the academic world to commercialized products [125]. However there are some label-free microfluidic systems commercially available such as the Triage system and the VIDAS platform, which use fluorescence for multiplex detection of proteins related to cardiac diseases [49]. An image of a microfluidic chip is shown in Figure 6.

#### 3.2.3. Fiber Optic Methods

Many research groups have developed fiber optic methods for biological sensing [105–112]. For example, a portable fiber optic fluorescence analyzer was used for determination of glomerular filtration rate of the kidney in animals [128]. King *et al.* have developed a device called the RAPTOR, a portable optical fluorimeter, that uses multiple single fiber optic probes to detect multiple analytes (one probe for each target) in a rapid and automated manner [51]. The RAPTOR has been shown to fluorescently detect spores and ovalbumin, among other bacterial, viral, and protein analytes, within 10 min, without any false positives, and without any sample processing (samples are added manually or via a computer-controlled air sampler) [124]. The authors also demonstrated the ability of the device to be reused with the same efficacy after undergoing several cycles of washing. This device improves upon previous optical biosensors such as the Analyte 2000, which connects single fiber optic probes in series to perform a multiplexed immunoassay [103] and the MANTIS, the precursor to the RAPTOR [51]. The RAPTOR can perform four sandwich immunoassays on the surface of waveguides in a field-deployable format where all processes, including data analysis, are automated [51,124]. Despite its benefits, there are some disadvantages of the RAPTOR, such as its size (18.6 cm × 27.4 cm × 17.3 cm) and weight (0.91 kg), both of which are larger than the ideal specifications for a portable POC device [51]. Additionally, the RAPTOR utilizes a separate fiber optic waveguide to detect each analyte, so each sample must be analyzed one at a time, and a total of only four analytes can be detected using its four different channels [124]. The next generation RAPTOR and the BioHawk, both sold by Research International, can detect up to eight analytes simultaneously, but each sample must be assayed individually [129]. The BioHawk design can potentially discern up to eight analytes and assess various samples simultaneously [56].

### 3.3. Current POC Sensors

While many scientists in academia and industry are researching and developing new POC tests, there are a few companies that have commercialized their products. Nova Biomedical is one company that has several products on the market ranging from hand-held devices to clinical analyzers as well as self-testing devices [130]. For example, this company has products that measure glucose, lactate, and creatinine. The glucose strip successfully removes hematocrit and other interferences and has been featured in several peer-reviewed publications [131–133].

Abbot Laboratories has a set of POC devices called i-STAT that are hand-held devices that can detect several biomarkers in patient samples using disposable cartridges [134]. The company sells several biomarker panels that can be used for diagnostic testing, such as a cardiac panel, a coagulation panel, a panel including many blood gases, a hematology panel, and a basic metabolic panel including electrolytes, glucose, and kidney biomarkers creatinine and blood urea nitrogen.

SenGenix is another company that is developing a new fluorescent approach to measuring biomarkers called fluorescently responsive sensors that bypass the need for lab-on-a-chip technology and use of a sandwich assay [135]. Instead, this company is engineering capture proteins that are conjugated to fluorescent molecules and simply require a light source to excite the fluorescent molecule and a camera to image the result.

The journal Nature, specifically, the Insights Supplement regarding Lab-on-a-Chip technology [136] highlighted some developments regarding lab-on-a-chip technology and their application to multiplexed POC medical diagnostics, For example, Yager, *et al.*, discuss the use of microfluidic technology for global health and note that successful POC tests created for use in the developing world must be designed for hot temperatures and may not be able to rely on refrigeration, reagents, power, or trained operators [137]. Taking into account the requirements of POC tests specific to global public health needs, some research groups are investigating the use of paper-based analytical devices [138–140]. Commercialized paper-based POC devices may be developed in the near future.

## 4. Real-World Applicability

The main theme of this review is the application of optical sensor platforms to study multiplexing capabilities for clinical use. POC testing has been demonstrated to not only reduce the amount of time it takes for physicians to make decisions regarding patient management, but also contributes to beneficial patient outcomes [141]. Proper implementation of testing at the point-of-need is also important for patient triage, such as in limited or low-resource settings, e.g., following an emergency, disaster, or other public health crisis [142]. To demonstrate the relevance of POC sensors to the field of medicine, we describe below three circumstances under which a hand-held device platform could be used to address real public health needs. There currently are none or few good-performing, commercialized products capable of multiplexing for these indications: (1) Measuring sexually transmitted infections (STIs) (2) measuring renal injury biomarkers associated with Acute Kidney Injury (AKI) and (3) measuring cardiac biomarkers, which is important for diagnosis and treatment of myocardial infarction [143]. In order for POC devices to be successfully implemented for these indications, they must be specific, sensitive, simple to use, reliable, capable of multiplexing, have reasonable cost, and have a low turn-around time [144].

### 4.1. Sexually Transmitted Diseases (STIs)

There is a demand for POC tests for to diagnose STIs because immediate diagnosis in a clinic can facilitate treatment and counseling, and decrease further transmission [24]. Since more than half of all sexually active people will contact some type of STI in their lifetime, and since many STIs are treatable, developing a POC test that could be used in a doctor's office would be beneficial. Even though rapid POC tests for STIs exist, they are not accurate enough by clinician's requirements, are difficult to read, or are too expensive. Typical STI tests include the wet mount test, a urine dipstick, and a rapid HIV test and typical turn-around time before results are available to healthcare providers and patients is 2–14 days [7]. Therefore, since current tests are not valuable, it is important for industry to create new tests that meet the end user's requirements. Clinicians have cited different limitations for current tests as compared to industry's perceptions. In order to create a useful POC test for STIs, industry needs to be more mindful of the needs of clinicians. For example, according to clinicians, the cost of the device set by the manufacturer is a more important factor than the amount of reimbursement received for performing the test. Furthermore, interruption of work flow and time frame required for the test are issues clinicians rated as more important than industry surmises. New tests need to be more sensitive, have good specificity, of reasonable cost, have a low turn-around time (5–20 min), be non-invasive, and easy to use and read [7,24]. Conducting tests at home rather than at a clinic face obstacles as some patients may not trust a test performed at home and may have trouble following instructions, especially if instructions are only provided in English. Gonorrhea and chlamydia seem to be the two priority areas for STI POC test development, followed by herpes simplex virus and seroconversion for HIV [7,24,145]. The first company that creates a multiplex POC device to probe for the top few STIs in a format similar to the home-use pregnancy test, often cited as the model prototype, will surely be able to make a great impact on public health.

### 4.2. Acute Kidney Injury Biomarkers

Measuring relatively new biomarkers such as Kidney Injury Marker-1 (KIM-1) and Neutrophil Gelatinase-Associated Lipocalin (NGAL) may improve personalized medicine by diagnosing patients correctly and quickly (earlier than existing methods allow) so that treatment can be administered based on the particular needs of the patient [146–148]. Current tests for diagnosing AKI involve measuring blood urea nitrogen (BUN) and serum creatinine, but these biomarkers are typically not elevated until 50% of kidney function is gone, which is clearly not adequate for reducing or preventing AKI [149]. Diagnosing AKI at the POC using a small amount of sample is important in a number of relevant situations. For example, a POC device could be used in the scenario of a biological attack where the lipopolysaccharide (LPS) of gram-negative bacteria could elicit an innate immune response [35,102]. Exposure to LPS (endotoxin) can cause systemic vasodilation and decreased renal perfusion and subsequent AKI [102]. Additionally, the resulting inflammation and cytokine release following exposure to the toxin will likely cause a nephrotoxic effect, leading to elevated levels of NGAL and KIM-1, which could indicate early kidney damage [35]. In another example, a POC device is useful in the developing world, where AKI is a major medical complication particularly with regard to sepsis, diarrheal illnesses, and infectious diseases. In this environment, low-resource medical tests are needed. A third scenario where a POC device would be useful for AKI diagnosis is following crash injuries and natural disasters such as earthquakes, which could adversely affect kidneys. Lastly, rapid diagnosis of AKI is important because early diagnosis, which can lead to quickly-instituted treatment, may results in more favorable outcomes for the patient. In these examples, testing and treating patients rapidly is of the utmost importance, and providing care to a large population using minimal resources can be facilitated by use of a POC device capable of multiplexing and using small samples.

### 4.3. Cardiac Biomarkers

While many diseases are so complex that examining multiple biomarkers is better than examining a single protein, in a study evaluating the use of three cardiac biomarkers, cardiac troponin (c Tnl), creatinine kinase isozyme MB (CK-MB) and myoglobin, it was found that use of c Tnl alone in a POC test was sufficient to diagnose chest pain and prevent myocardial infarction [150]. Use of cardiac troponin in POC tests has such high diagnostic accuracy (100% sensitivity and almost 100% specificity) that evaluating the other two biomarkers may not provide additional information, especially for patients with renal dysfunction [151,152]. In fact, c Tnl is such a strong biomarker to aid in diagnosis and treatment of myocardial infarction that current medical guidelines state that results of c TNL must be available to clinicians within 60 min after drawing the patient's blood [143]. However, use of these three biomarkers may be helpful in identifying patients with acute coronary syndrome who may be discharged from the hospital within 2 h [153] and use of CK-MB may help diagnose and treat myocardial infarction [154]. Since early diagnosis of myocardial infarction and other cardiac diseases can have a great impact on a patient's outcome, use of a POC sensor containing either simply c Tnl or c Tnl in conjunction with myoglobin and CK-MG would be very useful for different circumstances when adopted by hospitals around the world for use on a routine basis.

## 5. Conclusions and Outlook

Various types of optical sensing methods from ELISA and flow cytometry have been long been used for measuring proteins for clinical purposes. However, hand-held, field-deployable POC biosensors are predicted to revolutionize patient care by allowing quicker detection of proteins in a multiplexed fashion. These analytical tools may be useful in the detection of protein analytes that are critical for diagnosing renal and cardiac diseases, among many others. As the fields of proteomics, microfluidics and nanotechnology develop, assay formats will become simpler and more accessible. Use of POC devices to meet a variety of clinical needs such as diagnosing diseases, determining prognosis, and selecting the best therapy, is on the horizon.
